# Comparison of Maternal and Umbilical Cord Blood Selenium Levels in Low and Normal Birth Weight Neonates

**Published:** 2015-09

**Authors:** Lyly Nazemi, Mamak Shariat, Maryam Chamari, Reza Chahardoli, Leila Asgarzadeh, Fariba Seighali

**Affiliations:** 1 Maternal, Fetal and Neonatal Research Center AND School of Nutritional Sciences and Dietetic, Department of Clinical Nutrition, Tehran University of Medical Sciences, Tehran, Iran; 2 Maternal, Fetal and Neonatal Research Center, Tehran University of Medical Sciences, Tehran, Iran; 3 School of Nutritional Sciences and Dietetic, Department of Clinical Nutrition, Tehran University of Medical Sciences, Tehran, Iran; 4 Department of Cellular and Molecular, School of Nutritional Sciences and Dietetic, Tehran University of Medical Sciences, Tehran, Iran; 5 Breastfeeding Research Center, Tehran University of Medical Sciences, Tehran-Iran; 6 Blood Transfusion Research Center, High Institute for Research and Education in Transfusion Medicine AND Fetal and Neonatal Research Center, Tehran University of Medical Sciences Tehran, Iran

**Keywords:** Selenium level, maternal blood, umbilical cord blood, LBW neonates, Normal birth weight neonates

## Abstract

**Objective:** To compare the maternal and umbilical cord serum selenium concentrations in Low and normal birth weight neonates.

**Materials and methods:** A case-control study was carried out in Vali-Asr and Akbarabadi Hospitals (Jan. to Dec. 2013). Two groups; case group; 91 mothers who delivered a low birth weight (LBW) neonate and control group; 86 subjects who delivered a normal birth weight neonate were selected. Immediately after birth, 5 ml of maternal blood and umbilical cord blood were collected, and sent to laboratory to assay Se concentrations. To compare both groups' blood Se concentration, data were analyzed in SPSS 16.0.

**Results:** Eighty six (48.6%) mothers with normal birth weight neonates and 91 (51.4%) mothers with low birth weight infants entered the study. Mean maternal mothers' age and mean maternal blood Se were 28.55 + 5.90 years and 79.3756+26.46915. A significant association was seen between maternal blood and cord blood Se level in control and case group (P value < 0.0001, r = 0.69) and (P value<0.001, r = 0.79). On the other hand no differences were seen between 2 groups' maternal blood Se level (P Value = 0.65). Umbilical Cord blood Se concentration was not also different between case and control group (P value = 0.46).

**Conclusion:** We found that maternal and umbilical cord blood Se concentrations were not different in low and adequate birth weight infants, however; umbilical cord Se concentrations were positively correlated with maternal blood Se concentrations.

## Introduction

Selenium (Se) is a trace element that plays an important role in many humans' biochemical functions (1). Seleniumis known as a cofactor in major enzymes like glutathione peroxides (GSH-Px) and iodothyronine 5-deiodinase. It also plays a role in the antioxidant system by protecting cell membranes, and neutralizing the deleterious effects of free radicals (2).

The recommended daily intake of selenium is 50–200 µg. A significant correlation is seen between selenium dietary intake and its concentration in whole blood (3). Se and other micronutrient interventions during prenatal period can improve neonatal birth weight and decrease early infant mortality rate (4). Low Selenium concentrations in the mother or her fetus can affect on infection risk, a major cause of preterm birth (5). Selenium status is also important in the group of hospitalized neonates and infants. Different factors such as gestation age, habitat, mother's age and nutritional index can affect on the selenium concentration in maternal blood, umbilical cord (UC) blood and placenta (3). The maternal transplacental transfer of Se to fetus is limited .Se is stored in fetal liver between 20th and 40th week. The average cord blood Se level is reported 35–107 µg/L related to some factors like selenium content of soil in the different geographic region, gestational age, and serum Se concentration after 36 weeks (6).

Many studies have demonstrated selenium deficiency in several obstetric and reproductive complications like preterm deliveries and premature infants (6, 2). Bogden et al., found that low serum selenium concentrations in the first or second trimester of pregnancy can predict lower birth weight for full-term infants (7). Makhoul et al. Also showed a significant correlation between GA or birth weight and umbilical cord serum Se concentrations. On the other hand Tsuzuki et al. reported no relation between maternal serum Se concentration and birth weight (2, 4).

The status of selenium is important in the neonates and trace nutrient deficiency may play an important role in the development of some problems (2). There is limited data related to the selenium status in low birth infants particularly in Iran (6, 8). The aim of this study was to compare the maternal and umbilical cord serum Se concentrations in Low and normal birth weight neonates.

## Materials and methods

A case-control study was carried out in Vali-Asr and Akbarabadi Hospitals during January to December 2013. Two groups of mothers were selected for the study: case group; 91 mothers who delivered a low birth weight neonate (birth weight < 2500 gram) and control group; 86 subjects who delivered a normal birth weight neonate. The exclusion criteria were a past history of high risk pregnancy or systemic disease, prenatal Se supplements intake, twin or triplet delivery or any congenital malformations. 

Immediately after birth, 5 ml of maternal blood and 5 ml of umbilical cord blood (neonatal) were collected, labeled, stored at -20 ^0^C and sent to laboratory to assay mother's and neonate's blood Se level. 

All maternal and neonatal demographic data including mother's age, parity, blood Se concentration, occupation status, neonatal sex, weight, UC blood Se level, were recorded in questionnaires. Participants gave informed consent before entering the study.

To compare both groups' blood Se concentration, data were analyzed in SPSS 16.0. Independent T test, chi square and regression test were applied where applicable. The value of p < 0.05 was considered as level of significance.

No intervention was performed in our study and patients’ data were considered confidential. Ethics approval for the study was obtained from the institutional review board of Tehran University of Medical Sciences (ID; 15130-91-03-90).

## Results

Eighty six (48.6%) mothers with normal birth weight neonates and 91 (51.4%) mothers with low birth weight infant sentered the study. 149 (84.7%) mothers were unemployed and 87 mothers (49.2%) had diploma and higher education, Mean maternal mothers' age and mean maternal blood Se were 28.5+ 5.90 years and 79.3+ 26.46, respectively. Of 177 babies, 89 of them (50.3%) were male. Demographic data is shown in table 1. 

A significant association was seen between maternal blood and cord blood Se level in control group (P value < 0.0001, r = 0.69). This relation was also found in case group (P value < 0.001, r = 0.79). Our results showed that paired maternal-neonatal blood selenium concentrations were strongly correlated.

On the other hand no differences were seen between two groups' maternal blood Se level (P value = 0.65). UC blood Se concentration in two groups were not significantly different (P value = 0.46). Table 2 shows the variables among the two groups.

In case group, mothers' or cord blood Se concentration were not significantly correlated with parity, gestational age and birth weight. Details are shown in table 3. 

We could not find a significant correlation between maternal age and blood Se concentration, too (P value = 0.55).

## Discussion

In present study we compared the umbilical cord and maternal blood selenium concentrations in low and normal birth weight neonates. Based on our results, Mean umbilical cord Se levels in normal and low birth weight neonates' were 73.899 *±* 24.3798, 77.32 *±* 26.12 µg/ml that means in our country umbilical cord selenium is in normal range. These ranges can also be used as reference data besides other reports (3,9,10).In recent study the normal mean maternal serum Se concentration was 79.37 *±* 26.46 which is lower than reported by National Health and Nutrition Survey (123.6 µg/ml in 19-30 years old women) (8). This difference may be due to differences in Se contents in foods. 

We also found that Se levels in cord blood of both groups were lower than in their maternal blood, suggesting partially mother-fetus Se transfer (1).

Our study demonstrated a significant positive relationship between umbilical cord and maternal blood selenium concentrations. Our finding was consistent to Dobrzynski et al. study that has been shown strong correlations between Se concentrations in the blood components of the umbilical cord and maternal blood (11). Sun et al. also reported that the cord blood Se concentration was correlated with maternal Se (r = 0.29) (12).

We found no significant difference between maternal and umbilical Se concentrations in two groups with different birth weights. It is possible that other influencing factors mask or overpower the role of Se on neonatal birth weight. The relationship of birth weight with the mother and newborn's Se concentrations at birth was previously studied and confirmed no differences between appropriate and small-for-gestational-age newborn infants (2). Also Dobrzynskia et al. did not find significant correlations between birth weight and whole blood and plasma Se levels of cord blood as well (11). Contrary to our results, Tsuzuki showed that maternal serum Se concentration was an independent predictor of birth weight (4).

**Table 1:** Participants' demographic data

**Variables**		**Case group** **n = 91**	**Control group** **n = 86**
Maternal age (years)		28.41 ± 6.32	28.70 ± 5.44
Maternal blood Se (µg/ml)		80.69 ± 28.00	78.48 ± 25.54
Umbilical cord Se (µg/ml)		77.32 ± 26.12	73.89 ± 24.37
Gravida		1.98 ± 1.11	1.97 ± 1.44
Gestational age		28.82 ± 13.66	38.12 ± 0.91
Birth weight (g)		1923.90 ± 684.10	3337.79 ± 446.47
Male/female		42/48	47/35
Maternal weight before pregnancy (Kg)	58.45 ± 17.44		67.38 ± 21.13

**Table 2:** Comparison of maternal blood and umbilical cord Selenium Concentration

**Variables**	**Case group**	**Control group**	**P Value**
Maternalblood selenium level (µg/ml)	80.69 ± 28.00	78.48 ± 25.54	0.65
Cord blood levels of selenium (µg/ml)	77.32 ± 26.12	73.89 ± 24.37	0.46

**Table 3:** Relations between maternal or cord blood Se concentrations and some variables in case group

**Variables**	**Maternal blood Se (P value)**	**Umbilical cord Se (P value)**
Parity	0.051	0.345
Birthweight	0.961	0.660
Gestational age	0.502	0.915

There are some limitations to our study. Low selenium in early pregnancy may have a predictive value for fetal birth weight. Maternal Se serum concentrations were not measured in various gestational age-based groups that could provide valuable data. In addition, Se in the UC may be influenced by other factors like maternal weight or underlying disease that we did not consider in our study. We suggest more studies in different groups in different areas of Iran.

## Conclusion

We found that maternal and umbilical cord serum Se concentrations were not different in low and adequate birth weight infants. Additionally, maternal serum Se concentrations were positively correlated with umbilical cord Se concentrations.
